# Proximal Abduction Ulnar Osteotomy (PAUL): Short- and Long-Term Evaluation in Dogs Presenting Medial Compartment Disease

**DOI:** 10.3390/ani12040466

**Published:** 2022-02-14

**Authors:** Carolina Oliver Ballester, Carme Soler Canet, José Ignacio Redondo García, Nuria Fernández Salesa, Vicente Sifre Canet, Claudio Iván Serra Aguado

**Affiliations:** 1 Hospital Veterinario de Referencia UCV, Departamento de Medicina y Cirugía Animal, Facultad de Veterinaria y Ciencias Experimentales, Universidad Católica de Valencia San Vicente Mártir, 46018 Valencia, Spain; mdc.soler@ucv.es (C.S.C.); nuria.fernandez@ucv.es (N.F.S.); vj.sifre@ucv.es (V.S.C.); 2 Centro de Investigación Traslacional San Alberto Magno, Universidad Católica de Valencia San Vicente Mártir, 46002 Valencia, Spain; 3 Departamento Medicina y Cirugía Animal, Universidad Cardenal Herrera-CEU, CEU Universities, 46115 Valencia, Spain; nacho@uchceu.es

**Keywords:** developmental elbow disease, medial compartment disease, dogs, elbow arthroscopy, Proximal Abduction Ulnar Osteotomy (PAUL)

## Abstract

**Simple Summary:**

Developmental elbow disease is one of the main causes of lameness in the canine species and occurs often in large and giant breed dogs. The most frequent manifestation of this pathology is known as medial compartment disease due to a physiological overload in the medial region of the elbow. It is considered as a complex disease and the treatment is focused on relieving the pain and slowing the progression of osteoarthritis. The Proximal Abduction Ulnar Osteotomy (PAUL) technique is one of the newest techniques whose purpose is the transmission of loads from the medial to the lateral compartment. In this prospective case series, the authors use the combination of elbow arthroscopy and the PAUL technique and report a significant improvement in clinical signs, showing a low major complications rate with a high degree of owner satisfaction.

**Abstract:**

The aim of the study is to report the results obtained from performing a Proximal Abduction Ulnar Osteotomy (PAUL) technique in a cohort of dogs with medial compartment disease with short- and long-term follow-up, assessing the perception of the owners and describing the long-term complications associated with the technique. This is a clinical prospective study, including dogs diagnosed with medial compartment disease treated with elbow arthroscopy and PAUL between 2013 and 2020. Long-term follow-up data and postoperative complications were registered, and a questionnaire adapted from Fitzpatrick et al. 2009 was collected from owners. Thirty-three elbows in 26 dogs were included. The duration of follow-up ranged from 4 to 61 months (median: 24.76 months). At the end of the study, 73.1% of the owners reported excellent satisfaction and 74.1% of the owners would repeat the surgery in the same circumstances. The owner questionnaire showed a greater capacity to walk and run, without lameness and pain, and climb up and down stairs a year after surgery, being constant at the end of the study. Major postoperative complications were documented in 4/33 elbows (12.12%), including delayed union, implant failure, and osteophytosis of the medial aspect of the coronoid process. In conclusion, elbow arthroscopy and the PAUL technique achieved an evident improvement in the quality of life of patients with great satisfaction for most of the owners.

## 1. Introduction

The elbow joint is a complex structure formed by the humeral condyle, the trochlear notch of the ulna, and the radial head, which perfectly fit among them [1].

When an alteration happens in its development, this is known as developmental elbow disease, and it is one of the most common articular degenerative diseases and one of the main causes of lameness seen in the canine species [2,3,4,5]. This pathology is described as a polygenic, hereditary, developmental disease that occurs most often in large and giant breed dogs [2,3,4,6]. This complex disease includes four pathologies that, isolated or in combination, are described in developmental elbow disease: ununited anconeal process (NUPA), fragment/disease of the medial aspect of the coronoid process (MCD), osteochondrosis or osteochondritis dissecans (OCD) of the medial humeral condyle, and joint incongruence (radio–ulnar or ulnar–humeral) [1,2,5,7].

The most frequent manifestation of this developmental disease is called medial compartment disease (MCD) [8]. MCD is due to a physiological overload occurring in the medial coronoid process of the ulna and in the medial aspect of the humeral condyle; these findings have been detected by different studies at histomorphometric level and by means of computerized microtomography (microCT) [2,5,9,10]. The progression of this disease culminates in articular cartilage damage and subchondral bone overload involved in the MCD, with consequent fissures or fractures and erosion with subchondral bone exposure [2,4,6]. Dogs affected with MCD present a lameness of variable degree that is characterized by the abduction and external rotation of the limb during the advanced phase, evident pain to elbow manipulation, signs of variable synovial effusion, inflammation of the joint capsule, muscular atrophy, and decreased range of joint mobility [2,7].

Several diagnostic imaging techniques have been described: radiography, computed tomography, and arthroscopy [1,11,12]. A definitive diagnosis of developmental elbow disease can be reached combining these with the clinical signs. Each of these allows examination of different perspectives of joint structures, obtaining better global information about the condition of the elbow and helping to determine the most appropriate therapeutic option to improve prognosis factors [1,5,7,13].

Medial compartment disease can be treated by conservative or surgical management [14]. Treatment will vary depending on the age of the patient, the clinical signs, the degree of injury, the findings observed in the imaging tests, as well as the availability of the owner [7].

Today, several surgical techniques have been described whose purpose is based on modifying the anomalous distribution of joint loads; the most common load-shifting modifying osteotomy techniques are bioblique dynamic proximal ulnar osteotomy (BODPUO), sliding humeral osteotomy (SHO), and proximal abduction ulnar osteotomy (PAUL) [3,4,7,15,16,17]. The latter is one of the newest techniques, which was described by Ingo Pfeil who theorized that proximal osteotomy of the ulna fixed by a special plate would shift, abduct, and rotate the ulna, which would lead to lateralization of the paw, thus, unloading the medial compartment [3,16,18,19,20]. According to the current literature, this procedure seems to present favorable results in the short-term [18]. However, there are few studies that demonstrate the effectiveness of the treatment, and most of them are ex vivo studies [19,21]. A recent study describes the mid to long-term outcomes of arthroscopy and PAUL compared to arthroscopy alone; however, it is a retrospective study [22].

Therefore, the authors hypothesize a mid- and long-term improvement in lameness, pain, and clinical signs in dogs with medial compartment disease treated with elbow arthroscopy and PAUL technique, slowing the progression of the disease with a low complication rate. Therefore, the objective of this study is to report the results obtained from performing an elbow arthroscopy and PAUL technique in a cohort of dogs with medial compartment disease in the short- and long-term, assessing the perception of the owners and describing the long-term complications associated with the technique.

## 2. Materials and Methods

### 2.1. Case Selection

This is a prospective clinical study from 2013 to 2020. Patients diagnosed with medial compartment disease with compatible clinical signs, orthopedic examination, and imaging findings (elbow X-ray, CT scan, and arthroscopy) were enrolled prospectively in the study.

The study was approved by the Ethics Committee for Animal Welfare, in compliance with European guidelines 2010/63/EU. Dog owners were properly informed and gave their written consent.

Preoperatively, an orthopedic examination, lateral and cranio-caudal radiographic projections, and CT scan of the affected elbows were performed. All dogs underwent a combination of elbow arthroscopy and PAUL, uni- or bilaterally. In the case of bilateral elbow disease, the procedures were performed in two isolated procedures with a minimum period of 4 months between them. The Advanced Locking Plate System (ALPS) plate (Kyon, Boston, MA, USA) of 2- or 3-mm step varied according to the measurements made in the radiological imagen of the elbow. These measurements consisted of calculating the angle that forms the longitudinal axis of the radius with the axis of the elbow joint. Angulations greater than 82° require a 2-mm step, while angulations less than 82° require a 3-mm step [20].

Exclusion criteria were patients under 7 months of age, cartilage erosion that extended over most of the lateral aspect of the humeral condyle and radial head, and systemic illnesses or concurrent musculoskeletal disorders causing clinical signs at the time of surgery [3].

### 2.2. Surgical Procedure

The surgical procedure consisted of an elbow arthroscopy and PAUL, both performed in the same surgical procedure and by the same veterinary orthopedic surgeon. The anesthetic protocol used varied according to the clinical characteristics of each individual and the clinical preference of the anesthetist. Perioperative cephalosporine (22 mg/kg, IV, starting 30 min before approach and every 90 min during the surgical procedure) (Cefuroxima Normon^®^, Valencia, Spain) was administered. The patient was placed in dorsal recumbency for the elbow arthroscopy. The scope portal was placed 1 cm distal and caudal to the medial epicondyle of the humerus, and a 2.4 mm 30° scope was used (Karl Storz Endoscopy, Tuttlingen, Germany). An egress needle was placed in the caudomedial region of the elbow and the instrument portal was placed 1–2 cm cranial to the scope portal. This procedure allowed the assessment of the intra-articular structures and surgical treatment when needed. Medial coronoid fragments were extracted and subtotal coronoidectomy was performed via craniomedial triangulation; and the joints with cartilaginous erosions/ulcers were curetted until a healthy subchondral bone was obtained (using a curetted or shaver). With the same approach, the lateral compartment was assessed.

**Figure 1:**
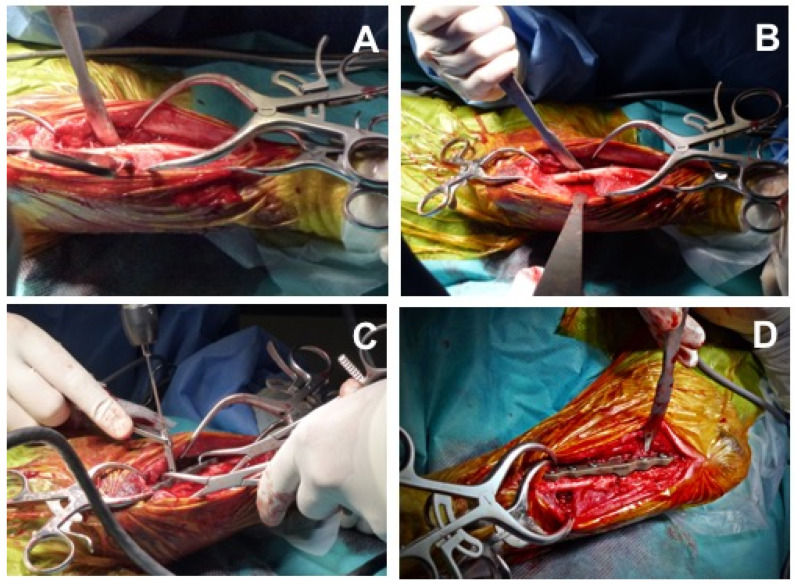
Surgical procedure: Proximal Abduction Ulnar Osteotomy (PAUL) technique. (**A**) Caudolateral approach at the level of the proximal third of the ulnar diaphysis. (**B**) Ulnar osteotomy in the proximal third of the ulnar diaphysis. (**C**,**D**) Location and fixation of the Advanced Locking Plate System (ALPS) plate in proximal and lateral ulna.

Subsequently, with the patient in the same position, a PAUL technique was performed, following the Kyon manufacturer’s instructions. A caudolateral approach was made at the level of the proximal third of the ulnar diaphysis. An ulnar osteotomy was performed and an Advanced Locking Plate System (ALPS) plate (Kyon, Boston, MA, USA) of 2- or 3-mm step was placed (Figure 1).

Postsurgical treatment consisted of robenacoxib (2 mg/kg, subcutaneously, every 24 h, for 2 days) (Onsior^®^, Basingstoke, United Kingdom), methadone (0.2 mg/kg, IV every 4 h, during the first 24–48 h) (metasedin^®^, Barcelona, Spain), buprenorphine (0.02 mg/kg, IV, every 12 h, for 4 days) (buprex^®^, Caldes de Montbui, Spain), according to the Glasgow pain scale [23], and omeprazole (1 mg/kg, IV, every 12 h, for 2 days) (Omeprazol^®^, Madrid, Spain). In addition, a Robert-Jones dressing was placed for the first 3 weeks with weekly rechecks and strict rest was advised for a month. The medical treatment was continued at home with robenacoxib (2 mg/kg, orally, every 24 h, for 4 weeks) and omeprazole (1 mg/kg, orally, every 12 h, for 4 weeks).

### 2.3. Short-Term Clinical Evaluation

All dogs were evaluated just before surgery and postoperatively at 1, 2, 3, and 8 weeks. At the first, second and third weeks after surgery, orthopedic exam with bandage change was performed, removing the bandage in the last one. At the 8-week recheck, all dogs underwent orthopedic examination and lateral and cranio-caudal radiographic projections of the elbow to assess an adequate positioning and integrity of the implant. Complications were reported in the short term.

### 2.4. Mid- to Long-Term Clinical Evaluation

Rechecks were made at 4 months, 12 months, and the end of study postoperatively. At each time, an orthopedic examination was performed. Furthermore, at 4 months postoperatively, lateral and cranio-caudal radiographic projections of the operated elbow were performed to assess a proper ossification and an adequate positioning and integrity of the implant. Complications were recorded as they were detected for mid- and long-term follow-up.

### 2.5. Short-, Mid- and Long-Term Questionnaire

A questionnaire was given to the owners, which consisted of evaluating different aspects of the physical activity of the patient as well as the degree of satisfaction of the owner.

All questionnaires referred to four specific moments: before surgery, 4 and 12 months after surgery, and at the end of the study.

The questionnaire used was adapted from Fitzpatrick et al. 2009 (Appendix A), in which the following issues were evaluated and categorized by a numerical scale from 0 to 10: (A) How well can your dog walk without pain? (B) How well can your dog run without pain? (C) How well can your dog climb up stairs? (D) How well can your dog climb down stairs? (E) What is your dog’s exercise tolerance? (F) How well can your dog sit down without pain or hesitation? (G) How well can your dog lie down without pain or hesitation? (H) How well can your dog rise on front legs without pain or hesitation? (I) Does your dog nod his/her head at walk? (J) Does your dog nod his/her head at run? (K) How would you grade the success of the operation? (L) Would you have this operation done again in the same circumstances?

For questions K and L, the animals were classified based on the score obtained by the owner.

On the one hand, for question K, a score of 9–10 was considered excellent satisfaction. A score of 7–8 was considered good satisfaction. A score of 5–6 was considered acceptable satisfaction, and a score less than 5 was considered poor satisfaction.

On the other hand, for question L, the owners would repeat the surgery in the same circumstances if the score was equal or greater than 7. Owners would not repeat the operation in the same circumstances if the score was less than 5. Scores 5 and 6 were considered doubtful.

### 2.6. Statistical Analysis

Data are described as median and range. R 4.0.3 (R Core Team, 2020) was used for analyzing the data. The variables over time were compared using two analyses. First, a Kruskal-Wallis test and a Wilcoxon rank sum test were performed to calculate pairwise comparisons between group levels with corrections for multiple testing. The Bonferroni method was used for adjusting *p* values. Second, a heteroscedastic one-way repeated measures ANOVA for trimmed means using the function rmanova of the WRS2 package (Mair and Wilcox, 2020) wasperformed. The level of statistical significance was set at *p* < 0.05 and the trimming level at 0.2 in both analyses [24,25].

## 3. Results

### 3.1. Case Selection

Thirty-three elbows (19 left, 14 right) in 26 dogs were treated by elbow arthroscopy and PAUL. Breeds included Labrador (6), Pitbull Terrier (4), Golden Retriever (3), German Shepherd dog (2), mixed (3), and 8 other breeds (1 each). There were 18 male and 8 female dogs with mean age at surgery of 27.52 months (interval: 7 to 119 months) and with an average weight of 33.18 kg (interval: 21.1 to 50.5 kg). Bilateral procedures were performed in seven dogs, and these were performed in two isolated procedures with an average of eight months (interval: 4 to 13 months) between them (Table 1).

Follow-up data were available for 26/26 dogs (33/33 elbows) at 4 months and 17/26 dogs (24/33 limbs) at 12 months: six of them were lost to follow-up (dogs 20, 22, 23, 25, 26 and 27, Table 1), one of them died in a traffic accident after the 4-month recheck (dog 11, Table 1), and in two of them the 4-month recheck was made at the end of study time and there was no long term follow-up available (dogs 32 and 33, Table 1).

Follow-up data were available for 18/26 dogs (22/33 limbs) at the end of the study: five of them were lost to follow-up (dogs 1–2, 3–4, 13–23, 6 and 19, Table 1), one was the dog who died in a traffic accident (dog 11, Table 1) and two of them were the dogs whose 4-month recheck coincided with the end of study time (dogs 32 and 33, Table 1).

### 3.2. Postoperative Owner Assessment

**Figure 2:**
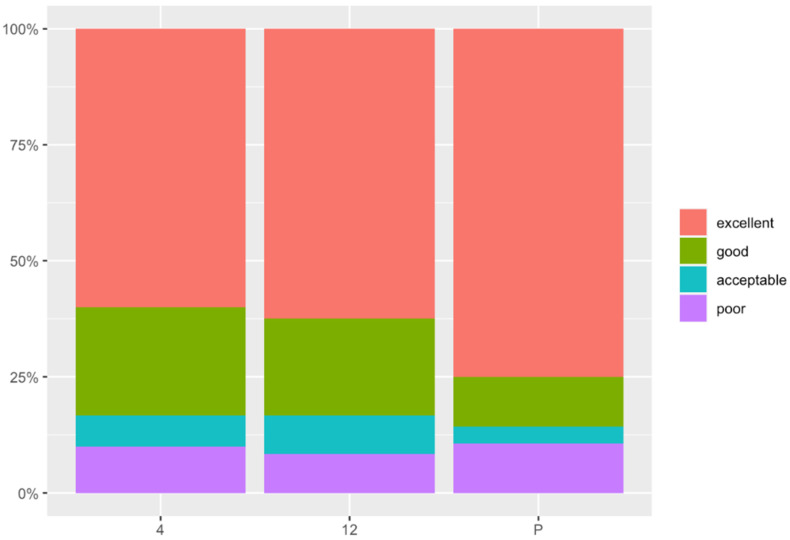
Degree of owner satisfaction at 4 and 12 months after surgery and at the end of the study (Question K). The X axis represents the time in months (4: four months after surgery; 12: twelve months after surgery; P: present). The Y axis represent the score, 0: poor and 10: excellent.

Two questions were asked to assess the degree of owner satisfaction and whether they would repeat the treatment in a similar situation:

At the end of the study, 73.1% of the owners showed excellent satisfaction, 11.5% of the owners showed good satisfaction, 3.8% of the owners showed acceptable satisfaction, and 11.5% showed poor satisfaction (Figure 2).

**Figure 3:**
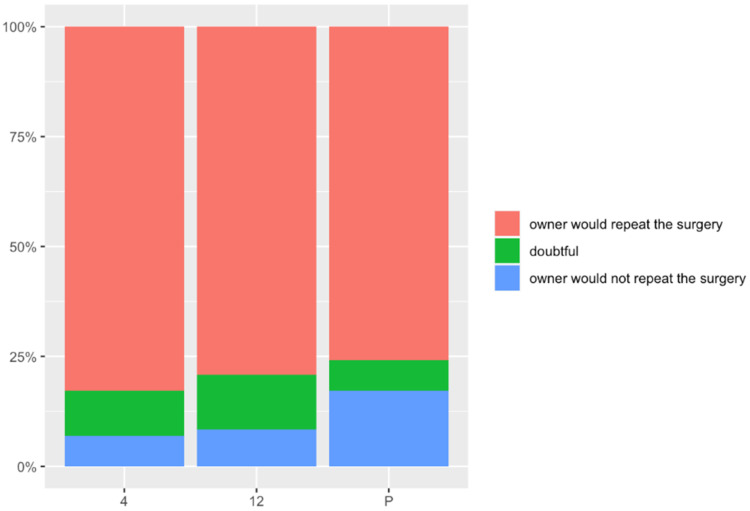
Answers to the question L at 4 and 12 months after surgery and at the end of the study: Would you have this operation done again in the same circumstances? The X axis represents the time in months (4: four months after surgery; 12: twelve months after surgery; P: present). The Y axis represent the score, 0: never and 10: definitely.

The results obtained from the question of whether the owners would repeat the surgery in a similar situation were that 74.1% would repeat the surgery, 18.5% would not, and 7.4% remained doubtful (Figure 3).

**Figure 4:**
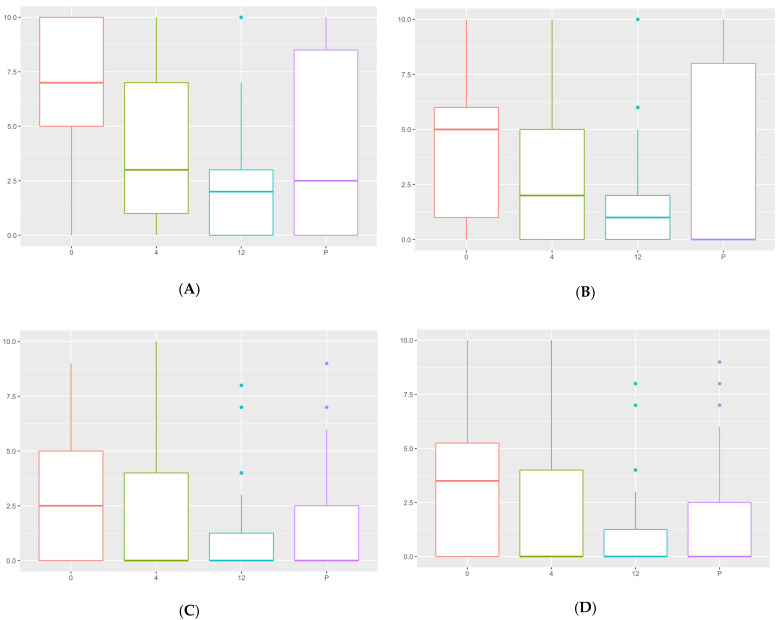
Comparative analysis of the capacity to walk, run, and climb up and down stairs. The X axis represents the time in months (0: prior to surgery; 4: four months after surgery; 12: twelve months after surgery; P: present). The Y axis represent the score, 0: not painful and 10: painful. (**A**) The capacity to walk without pain; (**B**) the capacity to run without pain; (**C**) the capacity to climb up stairs; and (**D**) the capacity to walk down stairs.

Ten other questions were asked regarding the patient’s ability to perform a specific activity (Table 2). The results were the following:

(A) How well can your dog walk without pain? (0: not painful/10: painful): mean value of 6.76 (SD: 3.29) before surgery, 3.85 (SD: 3.47) at 4 months, 2.29 (SD: 2.76) at 12 months after surgery, and 4.05 (SD: 4.09) at the end of the study. A significant improvement in lameness was observed at 4 and 12 months after surgery and at the end of the study, compared to before surgery (Figure 4).

(B) How well can your dog run without pain? (0: not painful/10: painful): mean value of 4.36 (SD: 3.51) before surgery, 2.81 (SD: 3.05) at 4 months, 1.57 (SD: 2.5) at 12 months after surgery, and 3.19 (SD: 4.06) at the end of the study. A significant improvement was observed between the time of surgery and 12 months after surgery (Figure 4).

(C) How well can your dog climb up stairs? (0: very well/10: poorly): mean value of 3 (SD: 2.92) before surgery, 2.13 (SD: 2.96) at 4 months, 1.29 (SD: 2.29) at 12 months after surgery, and 1.8 (SD: 2.89) at the end of the study. A significant improvement was observed at 12 months after surgery, compared to before surgery (Figure 4).

(D) How well can your dog climb down stairs? (0: very well/10: poorly): mean value of 3.31 (SD: 3.04) before surgery, 2.09 (SD: 3.02) at 4 months, 1.21 (SD: 2.23) at 12 months after surgery, and 1.95 (SD: 3.07) at the end of the study. Four and 12 months after surgery, there was a significantly greater ability to go downstairs without pain, compared to before surgery (Figure 4).

(E) What is your dog´s exercise tolerance? (0: copes fine with long walks/10: struggles on short walks): mean value of 3.82 (SD: 3.35) before surgery, 2.45 (SD: 3.07) at 4 months, 2 (SD: 2.54) at 12 months after surgery, and 2.76 (SD: 3.43) at the end of the study. This variable did not show changes over time.

(F) How well can your dog sit down without pain or hesitation? (0: very well/10: poorly): mean value of 1.55 (SD: 2.5) before surgery, 0.64 (SD: 1.85) at 4 months, 0.5 (SD: 1.22) at 12 months after surgery, and 1.45 (SD: 2.77) at the end of the study. No significant differences were observed before and after surgery regarding the ability to sit down without pain or hesitation.

(G) How well can your dog lie down without pain or hesitation? (0: very well/10: poorly): mean value of 1.55 (SD: 2.39) before surgery, 0.94 (SD: 2.33) at 4 months, 0.79 (SD: 1.93) at 12 months after surgery, and 2.27 (SD: 3.3) at the end of the study. No differences were shown when comparing the time before surgery with any other study time.

(H) How well can your dog rise on front legs without pain or hesitation? (0: very well/10: poorly): mean value of 3.42 (SD: 2.69) before surgery, 2.25 (SD: 2.34) at 4 months, 1.83 (SD: 1.93) at 12 months after surgery, and 2.27 (SD: 2.98) at the end of the study. At the end of the study there was a significantly greater ability to get up from the forelimbs without pain or hesitation when compared to the time before surgery. No other difference was shown in this variable.

(I) Does your dog nod his/her head at walk? (0: not at all/10: lots): mean value of 4.82 (SD: 3.43) before surgery, 3.53 (SD: 3.59) at 4 months, 2.83 (SD: 3.45) at 12 months after surgery, and 3.36 (SD: 3.57) at the end of the study. Four and 12 months after surgery there was a significantly greater ability to walk without moving the head, compared with time of surgery. No other difference was shown in this variable.

(J) Does your dog nod his/her head at run? (0: not at all/10: lots): mean value of 3.61 (SD: 3.89) before surgery, 2.78 (SD: 3.34) at 4 months, 1.17 (SD: 2.18) at 12 months after surgery, and 2.55 (SD: 3.49) at the end of the study. No differences were shown when comparing the time before surgery with any other study time.

(K) How would you grade the success of the operation? (0: poor/10: excellent): mean value of 8.13 (SD: 2.74) at 4 months, 8.38 (SD: 2.39) at 12 months after surgery, and 8.45 (SD: 2.95) at the end of the study. Four months after surgery, 57.1% of the owners showed excellent satisfaction, 25% showed good satisfaction, 7.1% showed acceptable satisfaction, and 10.7% showed poor satisfaction. Twelve months after surgery, 62.5% showed excellent satisfaction, 20.8% showed good satisfaction, 8.3% showed acceptable and poor satisfaction. At the end of the study, 73.1% showed excellent satisfaction, 11.5% showed good satisfaction, 3.8% showed acceptable satisfaction, and 11.5% showed poor satisfaction. No significant differences were observed during the study time.

(L) Would you have this operation done again in the same circumstances? (0: never/10: definitely): mean value of 8.16 (SD: 3.31) at 4 months, 8.54 (SD: 2.48) at 12 months after surgery, and 8.07 (SD: 3.15) at the end of the study. Four months after surgery, 81.5% would repeat the surgery in a similar circumstance, 7.4% would not, and 11.1% remained doubtful. Twelve months after surgery, 79.2% would repeat the surgery, 8.3% would not, and 12.5% remained doubtful. At the end of the study, 74.1% would repeat the surgery, 18.5% would not, and 7.4% remained doubtful. No significant differences were observed during the study time.

### 3.3. Radiographic Outcomes

**Figure 5:**
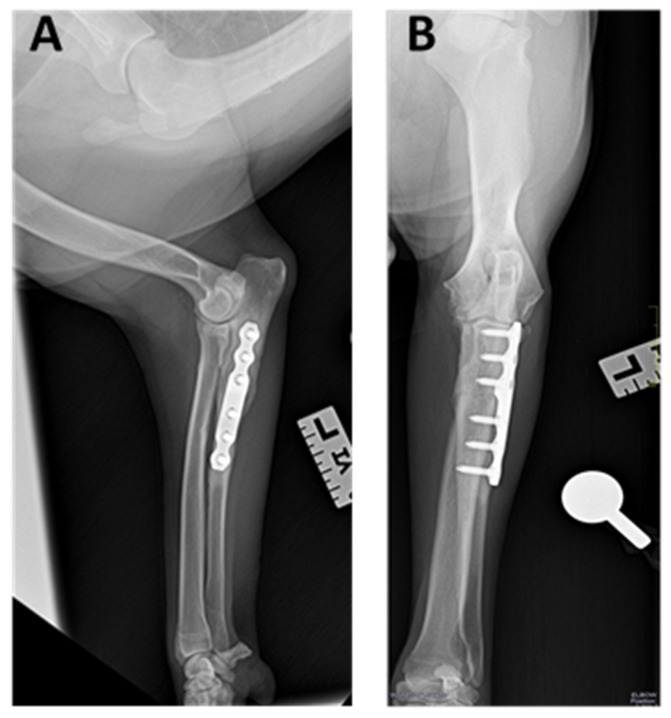
(**A**) Lateral and (**B**) cranio-caudal projection of the left elbow at 4 months after surgery. Radiographic evidence of union and bone remodeling between proximal and distal segments (dog 16, Table 1).

**Figure 6:**
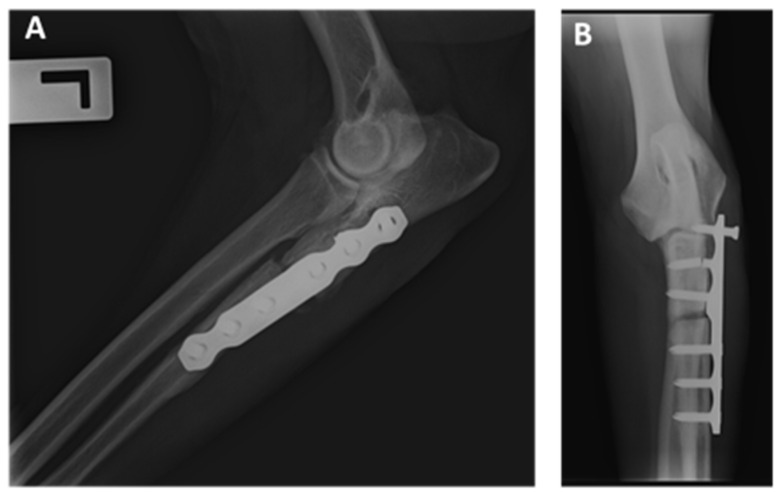
(**A**) Lateral and (**B**) cranio-caudal projection of the left elbow at 4 months after surgery. Delayed union and osteolysis due to the instability were identified. At the same time, the caudal placement of the plate in the ulna lead to greater stress on these implants (dog 12, Table 1).

Radiographic evidence of union and bone remodeling between proximal and distal segments was documented for 31/33 ulnar at 16 postoperative weeks (Figure 5). Two dogs presented a delayed union and osteolysis due to instability of the osteotomy: one of them resolved with re-intervention (dog 23, Table 1); and the other one was re-operated to remove the implant without subsequent stabilization due to economic constraints (dog 12, Table 1) (Figure 6).

### 3.4. Minor and Major Complications

Seven elbows (21.21%) presented postoperative complications. Minor complications were seen in 3/33 elbows (9.09%): one dog presented flexor carpi ulnaris muscle contracture, without functional consequences (dog 21, Table 1); and two dogs had a screw break, resolved with conservative treatment (dogs 15 and 29, Table 1).

Major complications were observed in 4/33 elbows (12.12%): one dog had pain on elbow flexion and extension which resolved after the implant removal (dog 24, Table 1); two dogs presented screw migration due to delayed union and osteolysis secondary to instability at the osteotomy site (Figure 5), both were re-operated and one of them without subsequent stabilization by economic constraints (dogs 12 and 23, Table 1); the last dog presented a fragmentation of the medial coronoid process 7 months after surgery, so fragment and implant removal was performed at the same time (dog 30, Table 1).

## 4. Discussion

Our hypothesis was accepted, because the long-term outcome with the combination of elbow arthroscopy and PAUL reported a significant improvement in lameness with a lower incidence of long-term re-intervention in our study.

Elbow arthroscopy is usually performed to remove the fragment of MCP or perform a subtotal coronoidectomy [26,27,28]. The short- and long-term outcomes after arthroscopic treatment in dogs with MCD of the elbow provide objective evidence of long-term improvement in function [4]. However, another study reported a progressive OA and cartilage damage 2.7 years later after arthroscopic treatment alone [29]. In contrast, the combination of elbow arthroscopy and PAUL reported a significant improvement in lameness with a lower incidence of long-term re-intervention.

A recent retrospective study by Coguill et al. showed no significant benefit of elbow arthroscopy and PAUL procedure over elbow arthroscopy alone for dogs with MCD using the Canine Brief Pain Inventory Score [22]. This could be due to the fact that the cases selected in the Coguill study had a Modified Outerbridge score >3. In contrast, in the present study authors included patients with a lower score. Furthermore, the Coguill study had limitations in regard to the small sample size, the retrospective and subjective nature, and the variability in the surgical execution between surgeons, which could influence the outcome and the incidence of complications.

There are some surgical techniques available whose purpose is to modify the anomalous distribution of joint loads from the medial compartment in favor of the lateral compartment, the most commonly used are the BODPUO, SHO, and PAUL techniques [1,3,4,7,16,17].

The BODPUO technique is indicated in dogs with moderate to severe medial coronoid process disease, improving elbow congruity and decreasing the progression of osteoarthritis. However, this treatment is indicated in dogs up to skeletal maturity, between 5 and 12 months of age, since in mature dogs, this technique increases the morbidity and prolonged healing time [30]. However, the PAUL technique can also be used in skeletally mature animals whose purpose is to alleviate pressure in the medial compartment [16].

The SHO technique showed a significant improvement in ground reaction forces in 67% of dogs by 6 months, increasing to 90% by 12 months but decreasing to 43% by >12 months, showing a highly variable recovery time. However, 90% of owners detected less lameness at the final evaluation compared with preoperative lameness [3]. Whereas, in the current study, 62.5% of the owners reported excellent satisfaction by 12 months, increasing to 73.1% by >12 months, with the combination of elbow arthroscopy and PAUL technique. On the other hand, the complication rate for the SHO technique was 17%, describing cases with infection and implant failure as major complications [3]. However, the current study described major complications in 12.12% (4/33) of elbows, where three of them resolved with the second surgery [3].

PAUL is indicated in adult patients diagnosed with an MCD that does not respond to conservative treatment or elbow arthroscopy alone [31]. At the same time, the use of elbow arthroscopy as an adjunctive therapy in PAUL technique, helps confirm the MCD, allowing removal of the medial coronoid fragment, coronoidectomy, and OCD treatment and assessment of the integrity of the lateral compartment [26,29]. However, in the authors’ opinion, the PAUL technique should not be used in the following cases: (1) the final stages of osteoarthrosis, where proliferative changes prevent the correct performance of the technique, (2) humerus–ulnar incongruity, since the PAUL technique would not achieve the desired load change, and (3) cases with disease in the lateral compartment that advises against transporting loads to it [32].

The owner questionnaire comparative analysis showed a greater capacity to walk and run, without lameness and pain, and climb up and downstairs a year after surgery, being constant at the end of the study (Figure 4). This could mean the maximum effect of the combination of elbow arthroscopy and PAUL technique is achieved at 12 months after surgery being constant in time, probably by modulating or slowing the progression of the osteoarthritis [31].

The results also showed a significant improvement according to walking and rise on forelimbs without pain or hesitation at the end of the study, compared to before surgery.

However, no significant differences were observed before and after surgery regarding the dog’s exercise tolerance, the ability to sit down and lie down without pain or hesitation, and to nod his/her head at run. This could be due to the degree of osteoarthritis prior to surgery and the patient habits to sit and lie down.

At the end of the study, 73.1% of owners reported excellent satisfaction (with a score >9), and 74.1% of the owners would repeat the surgery in a similar situation, and both variables remained stable over time. In total, 11.5% of the owners were not satisfied with the intervention, and 18.5% would not repeat the surgery. This coincides with the postsurgical complications observed in their pets, where four dogs were re-operated on surgically, two of them due to delayed union of the osteotomy site with associated osteolysis, one due to reduced range of motion of the elbow with associated pain, and one for the presence of an osteophyte in the medial coronoid process, which led to a new fragment into the joint.

A recent retrospective study described complications as fairly common after PAUL, particularly in heavier dogs, showing as a major complication rate 17.56%, including nonunion, implant failure, and infection. However, the technique was performed by several surgeons with varying levels of experience, so the low experience of some surgeons can increase the number of postoperative complications and the number of potential technical errors identified on postoperative radiographs [33].

Finally, the present study has some limitations. Firstly, this was due to the small sample size, the lack of an objective evaluation with force plate analysis of the lameness at different times of the study, the loss of data over time, and the effect on the outcome of the use of the 2 or 3 mm step plate. Additionally, there was a lack of a control group to compare the results obtained with the combined therapy by arthroscopy and PAUL. Furthermore, there are some factors not evaluated that could affect the results, such us the lack of use of the modified Outerbridge score, the severity and duration of lameness at the time of presentation, the cartilage damage degree and osteoarthritis (OA) grade presented, since all of them could affect the outcome and prognosis [34]. Finally, another limitation, as for any study on surgical techniques, is the variability in the surgical execution, which could influence the outcome and the incidence of complications.

## 5. Conclusions

In conclusion, the combination of elbow arthroscopy and PAUL technique ameliorated lameness and pain associated with MCD at short- and long-term follow-up, with 73.1% of the owners showing excellent satisfaction. The satisfaction was mainly due to the significant improvement in their dog’s capacity to walk without pain. The major complication rate was 12.12% (4/33 dogs) however, only two dogs presented a major complication due to a delayed union and osteolysis secondary to instability at the osteotomy site.

Nevertheless, more investigation is necessary with a control group, a large sample size, and objective gait analysis at different times of the study to achieve more conclusive results in patients undergoing elbow arthroscopy and PAUL technique for MCD.

## Figures and Tables

**Table 1:** Descriptive results of 33 elbows in 26 dogs treated by arthroscopy and PAUL.

Case	Breed	Sex	Weight (Kg)	Forelimb	Age Surgery (Months)	Size Plate (Number)	Plate Step (mm)	Follow-up (Months)
1	German Shepherd	Female	27	Right	10	9	3	12
2	German Shepherd	Female	32	Left	14	10	3	12
3	Labrador Retriever	Male	23	Right	10	9	3	12
4	Labrador Retriever	Male	26	Left	14	9	3	12
5	Pitbull Terrier	Female	32	Left	30	9	3	61
6	Golden Retriever	Male	30	Left	18	9	3	12
7	American Bulldog	Male	43	Right	24	10	3	55
8	Newfoundland	Male	49	Right	13	11	3	57
9	Newfoundland	Male	50.5	Left	20	11	3	51
10	Pitbull Terrier	Female	24	Right	10	9	3	53
11	Mixed	Male	32.4	Left	7	10	3	4
12	Australian Shepherd	Male	41	Left	7	10	2	39
13	Labrador Retriever	Male	35.8	Left	11	10	2	12
14	Golden Retriever	Male	33	Left	11	10	2	34
15	Mixed	Female	22.6	Right	7	9	2	33
16	Pitbull Terrier	Male	32	Right	7	10	2	33
17	Pitbull Terrier	Male	24	Right	108	9	2	33
18	Bernese Mountain	Male	37	Right	18	10	2	33
19	Pitbull Terrier	Male	30.5	Left	11	10	2	30
20	Argentin Dogo	Female	30	Right	18	9	2	28
21	Rottweiler	Female	38	Left	24	10	2	27
22	Mixed	Female	27	Left	12	9	2	26
23	Labrador Retriever	Male	35.8	Right	24	10	2	4
24	Mixed	Female	21.1	Left	36	9	2	21
25	German Shepherd	Male	26.4	Right	43	9	2	20
26	Pitbull Terrier	Male	24	Left	119	9	2	20
27	Labrador Retriever	Male	39.7	Right	60	10	2	19
28	Giant Schnauzer	Male	43.1	Left	24	11	2	16
29	Labrador Retriever	Female	32	Left	84	9	2	14
30	Labrador Retriever	Male	35	Left	30	10	2	14
31	Golden Retriever	Male	41	Left	24	10	2	12
32	Labrador Retriever	Male	42	Right	48	10	2	4
33	Caucasian Shepherd	Male	35	Left	12	10	2	4

**Table 2:** Comparative results between variables 0 (prior to surgery), 4 and 12 months after surgery, and at the end of the study.

	T0 vs. T4	T0 vs. T12	T0 vs. TP	T4 vs. T12	T4 vs. TP	T12 vs. TP
A	<0.01	<0.01	<0.01	0.06	0.57	0.36
B	0.11	<0.01	0.45	0.08	0.77	0.51
C	0.13	<0.05	0.19	0.1	0.68	0.32
D	<0.05	<0.05	0.08	0.25	0.4	0.27
E	0.08	0.06	0.18	0.66	0.44	0.74
F	0.05	0.36	0.5	0.63	0.34	0.3
G	0.08	0.3	0.51	0.67	0.23	0.34
H	0.06	0.06	<0.05	1	0.91	0.92
I	<0.05	<0.05	0.08	0.2	0.53	0.5
J	0.15	0.14	0.41	0.08	0.53	0.42
K	-	-	-	0.08	0.53	0.42
L	-	-	-	0.75	0.75	0.37

## Data Availability

The datasets used and/or analyzed during the current study are available from the corresponding author on reasonable request.

## Appendix A

Owner questionnaire for Proximal Abducting Ulnar Osteotomy (PAUL).

Animal name:	Forelimb:
Surname:	Data surgical:
Breed:	Age at surgery:
Weight:	Size of the plate:
Sex:	Plate step:

Please answer the following questions by marking on the line how well or poorly your pet can perform each of the following.

	**Time 0 (before Surgery)**	**Time 4 Months (after Surgery)**	**Time 12 Months (after Surgery)**	**Present**
How well can your dog walk without pain? 0: not painful 10: painful				
How well can your dog run without pain? 0: not painful 10: painful				
How well can your dog climb UP stairs? 0: very well 10: poorly				
How well can your dog climb DOWN stairs? 0: very well 10: poorly				
What is your dog´s exercise tolerance (ability to go for walks without stopping or tiring)? 0: Copes fine with long walks 10: Struggles on short walks				
How well can your dog sit down without pain or hesitation 0: very well 10: poorly				
How well can your dog lie down without pain or hesitation? 0: very well 10: poorly				
How well can your dog rise on front legs without pain or hesitation? 0: very well 10: poorly				
Does your dog nod his/her head at walk? 0: not at all 10: lots				
Does your dog nod his/her head at run? 0: not at all 10: lots				
How would you grade the success of the operation? 0: poor 10: excellent				
Would you have this operation done again in the same circunstances? 0: never 10: definitely				
